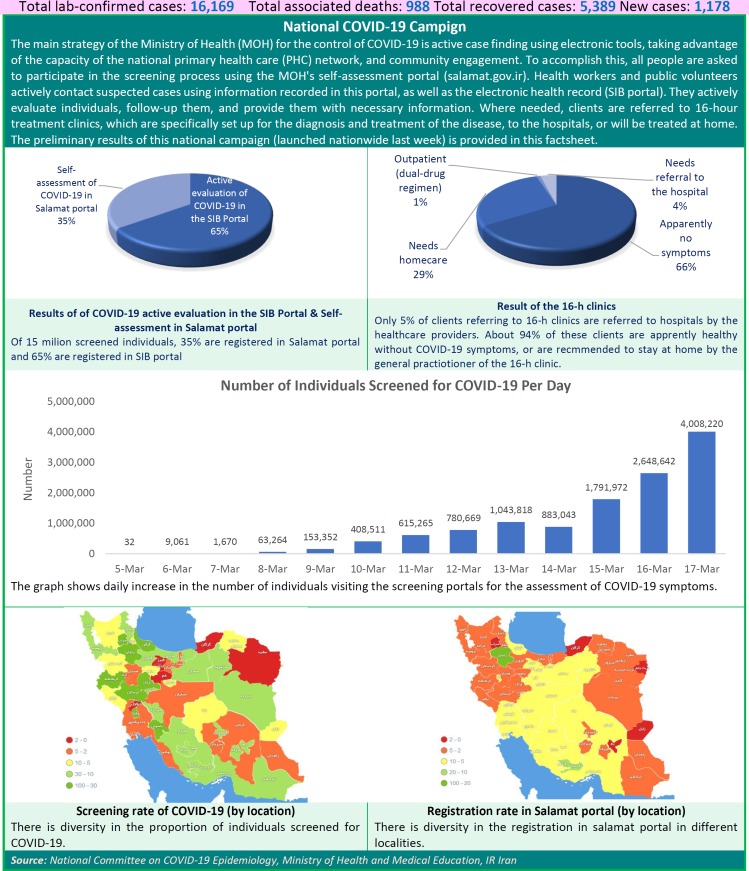# Daily Situation Report on Coronavirus disease (COVID-19) in Iran; March 17, 2020

**Published:** 2020-03-17

**Authors:** National Committee on COVID-19 Epidemiology, Ministry of Health and Medical Education, IR Iran

**Keywords:** COVID-19, severe acute respiratory syndrome coronavirus 2, Hospital Mortality, epidemiology, Pandemics, Health Information Exchange

## Abstract

The main strategy of the Ministry of Health (MOH) for the control of COVID-19 is active case finding using electronic tools, taking advantage of the capacity of the national primary health care (PHC) network, and community engagement. To accomplish this, all people are asked to participate in the screening process using the MOH's self-assessment portal (salamat.gov.ir). Health workers and public volunteers actively contact suspected cases using information recorded in this portal, as well as the electronic health record (SIB portal). They actively evaluate individuals, follow-up them, and provide them with necessary information. Where needed, clients are referred to 16-hour treatment clinics, which are specifically set up for the diagnosis and treatment of the disease, to the hospitals, or will be treated at home. The preliminary results of this national campaign (launched nationwide last week) is provided in this factsheet.

**Figure:**